# Surgical Challenges in Infective Endocarditis: State of the Art

**DOI:** 10.3390/jcm12185891

**Published:** 2023-09-11

**Authors:** Alessandra Iaccarino, Alessandro Barbone, Alessio Basciu, Enea Cuko, Ginevra Droandi, Denise Galbiati, Giorgio Romano, Enrico Citterio, Andrea Fumero, Iside Scarfò, Rossella Manzo, Giovanni La Canna, Lucia Torracca

**Affiliations:** 1 Cardiovascular Department, UO of Cardiac Surgery of IRCCS Humanitas Research Hospital, 20089 Rozzano, Italy; alessandro.barbone@humanitas.it (A.B.); alessio.basciu@humanitas.it (A.B.); enea.cuko@humanitas.it (E.C.); ginevra.droandi@humanitas.it (G.D.); denise.galbiati@humanitas.it (D.G.); xgiorgioromano@gmail.com (G.R.); enrico.citterio@humanitas.it (E.C.); andrea.fumero@humanitas.it (A.F.); lucia.torracca@humanitas.it (L.T.); 2 Cardiovascular Department, Applied Diagnostic Echocardiography of IRCCS Humanitas Research Hospital, 20089 Rozzano, Italy; iside.scarfo@humanitas.it (I.S.); rossella.manzo@humanitas.it (R.M.); giovanni.lacanna@humanitas.it (G.L.C.)

**Keywords:** infective endocarditis, native valve endocarditis, prosthetic valve endocarditis, mitral valve repair, tricuspid valve repair, aortic mitral curtain, Commando procedure

## Abstract

Infective endocarditis (IE) is still a life-threatening disease with frequent lethal outcomes despite the profound changes in its clinical, microbiological, imaging, and therapeutic profiles. Nowadays, the scenario for IE has changed since rheumatic fever has declined, but on the other hand, multiple aspects, such as elderly populations, cardiovascular device implantation procedures, and better use of multiple imaging modalities and multidisciplinary care, have increased, leading to escalations in diagnosis. Since the ESC and AHA Guidelines have been released, specific aspects of diagnostic and therapeutic management have been clarified to provide better and faster diagnosis and prognosis. Surgical treatment is required in approximately half of patients with IE in order to avoid progressive heart failure, irreversible structural damage in the case of uncontrolled infection, and the prevention of embolism. The timing of surgery has been one of the main aspects discussed, identifying cases in which surgery needs to be performed on an emergency (within 24 h) or urgent (within 7 days) basis, irrespective of the duration of antibiotic treatment, or cases where surgery can be postponed to allow a brief period of antibiotic treatment under careful clinical and echocardiographic observation. Mainly, guidelines put emphasis on the importance of an endocarditis team in the handling of systemic complications and how they affect the timing of surgery and perioperative management. Neurological complications, acute renal failure, splenic or musculoskeletal manifestations, or infections determined by multiresistant microorganisms or fungi can affect long-term prognosis and survival. Not to be outdone, anatomical and surgical factors, such as the presence of native or prosthetic valve endocarditis, a repair strategy when feasible, anatomical extension and disruption in the case of an annular abscess (mitral valve annulus, aortic mitral curtain, aortic root, and annulus), and the choice of prosthesis and conduits, can be equally crucial. It can be hard for surgeons to maneuver between correct pre-operative planning and facing unexpected obstacles during intraoperative management. The aim of this review is to provide an overview and analysis of a broad spectrum of specific surgical scenarios and how their challenging management can be essential to ensure better outcomes and prognoses.

## 1. Introduction

Infective endocarditis represents an infection involving specific cardiac tissues: the endocardium, native heart valves at any position, prosthetic heart valves, or any implanted cardiac devices. IE can be clinically insidious since epidemiology and natural history are in constant evolution, reflecting the complex interaction between an aging population, multiresistant microorganisms, evolving patterns of healthcare, available therapies, and the application of aggressive surgery. IE remains a relatively uncommon condition, with an annual occurrence of approximately 3–10 cases per 100,000 individuals [1]. The characteristics of this ailment exhibit variations across the globe, particularly when comparing countries with differing income levels. In regions with limited economic resources, rheumatic heart disease continues to be the prominent risk factor. Patients in these areas are typically younger, and the primary source of infection stems from penicillin-sensitive streptococci, commonly acquired within the community. Conversely, the prevalence of rheumatic heart disease has decreased in affluent nations due to enhanced living conditions and the widespread availability of antibiotics. In higher-income countries, the principal risk factors for IE encompass degenerative valve issues, diabetes, cancer, intravenous drug usage, and congenital heart abnormalities. Furthermore, this demographic skews older, with the average age transitioning from the mid-40s in the early 1980s to surpassing 70 years between 2001 and 2006 [2]. This changing epidemiology mirrors the wide medical advances both in terms of the rise in cardiovascular device implantation procedures (prosthetic valves, pacemaker leads, transcatheter valves, and vascular devices) and in terms of the better use of multiple imaging modalities and multidisciplinary care, which also translates into escalations in diagnosis. A diagnosis of infective endocarditis needs the integration of clinical findings, microbiological analyses, and imaging results. Due to its heterogeneity, the management of patients with IE necessitates a multidisciplinary approach with physicians, cardiologists, cardiac surgeons, electrophysiologists, microbiologists, histopathologists, infectious disease specialists, radiologists, and echocardiographers. Despite advancements in both detection and treatment, results continue to exhibit unsatisfactory trends, which is evidenced by in-patient mortality rates of 18%, along with a 6-month mortality rate of 30%. The impact on outcomes is significantly influenced by factors such as S. aureus infection, age progression, the persistence of positive blood cultures despite receiving suitable antibiotic treatment, and the existence of notable concurrent health conditions [1]. It is imperative to achieve a precise diagnosis promptly and administer antimicrobial treatment to mitigate the potential for complications and enhance the overall prognosis. Surgical intervention is carried out in approximately 40–50% of infective endocarditis cases [3] in order to avoid progressive heart failure, irreversible structural damage in case of uncontrolled infection, and the prevention of embolism. A resume of European and American guidelines is reported in Table 1.

American College of Cardiology/American Heart Association (ACC/AHA) and the European Society of Cardiology (ESC) guidelines widely focus on diagnostic and therapeutic management, surgical indications and patient selection, operative risk assessment, and timing of surgery in relation to specific systemic circumstances. Given the paucity of randomized or large-scale observational data in IE, we base our knowledge on a few large registries reporting, among other information, operative risk assessment in order to predict and improve the clinical outcomes of patients. Cohorts and registries, such as the International Collaboration on Endocarditis (ICE), Society of Thoracic Surgeons-Infective Endocarditis Cohort (STS-IE), and The European Society of Cardiology (ESC) EURObservational Research Programme (EORP) European Endocarditis (EURO-ENDO) registry, mainly relate operative risk and short-term outcomes to a higher and heterogeneous number of variables, including comorbid medical conditions, acute status, and systemic or embolic complications. The first systematic review and meta-analysis, derived from 11 studies, with a specific focus on prediction models of postoperative mortality in patients with IE, underlined 11 preoperative factors that are related to increased postoperative mortality: the presence of cardiogenic shock, NHYA class ≥ III, a need for urgent surgery, the presence of paravalvular abscess, preoperative renal failure, previous cardiac surgery, etiology (*S. aureus* and fungi), female sex, age, prosthetic valve IE, and multivalvular involvement [6]. It should be noted that this is the first analysis relating specific surgical characteristics (paravalvular abscess) and prognosis, closing, for the first time, a huge gap in research. Anatomical and surgical factors, such as the presence of native or prosthetic valve endocarditis, a repair strategy when feasible, anatomical extension and disruption in the case of an annular abscess (mitral valve annulus, aortic mitral curtain, aortic root, and annulus), and the choice of prosthesis and conduits, can be crucial and can affect prognosis. It can be hard for surgeons to maneuver between correct pre-operative planning and facing unexpected obstacles during intraoperative management. The aim of this review is to provide an overview and analysis of the broad spectrum of specific surgical scenarios and how their challenging management can be essential to ensure better outcomes and prognoses.

## 2. Native Valve Endocarditis: Repair versus Replacement

Native valve infective endocarditis is not frequently encountered, with an estimated occurrence of about 2 to 10 instances per 100,000 person-years [7]. Damage to the valvular endothelium or endocardium exposes subendothelial collagen and matrix molecules, prompting platelet and fibrin accumulation and forming a microthrombotic lesion. Subsequently, bacteria present in the bloodstream colonize this lesion, initiating replication and leading to the development of an infected vegetation, a defining characteristic of infective endocarditis. Certain cardiac conditions can predispose individuals to the onset of infective endocarditis, such as congenital anomalies (like ventricular septal defects and bicuspid aortic valves) or acquired valvular issues (including degenerative valvular diseases, aortic stenosis, and rheumatic heart disease). In more developed nations, the principal predisposing cardiac conditions encompass degenerative valvular disorders, congenital valvular irregularities, and the presence of intracardiac devices [8]. On the other hand, factors not directly related to the heart, such as poor dental health, intravenous drug usage, hemodialysis, chronic liver disease, diabetes, compromised immune function, neoplastic diseases, and the presence of indwelling intravascular devices, can also contribute to an individual’s susceptibility to this condition. The general principle of IE surgery is to obtain a radical debridement of vegetation and infected tissue and to avoid and limit, when possible, the use of foreign material. When it comes to infective endocarditis involving native heart valves, the mitral valve (MV) tends to be affected most frequently, accounting for approximately 40–50% of cases [9]. Throughout history, mitral valve repair (MVr) has demonstrated better survival rates and overall outcomes, especially in comparison to valve replacement (MVR), effectively restoring life expectancy across all age brackets. Furthermore, a strong association has been observed between surgical volume and patient outcomes, both at hospital and surgeon levels. This suggests that the frequency of interventions performed by a surgeon is an independent predictor of successful repair, surgical outcomes, and enhanced survival [10]. However, within the context of endocarditis, the advantages and predictors of mitral valve repair are less well established. Within this particularly challenging and intricate patient population, real-world statistics from multicenter reports indicate that rates of successful repair range only from 19% to 32% [11]. Numerous case reports and retrospective studies have presented positive surgical outcomes associated with mitral valve repair (MVr) for infective endocarditis (IE). Dreyfus et al. [12] were pioneers in demonstrating the feasibility of successful MVr for active native IE. The application of repair techniques eliminates the need to introduce a prosthetic material into infected tissue, curtails the spread of the infective process, and enhances the preservation of left ventricular function. It is worth noting that while the reported favorable outcomes may not precisely mirror real-world scenarios due to the limited case numbers and high degree of selectivity, multiple reviews have consistently highlighted the significantly superior results of MVr when compared to mitral valve replacement (MVR) in patients with IE. However, despite these positive trends, a standardized approach to the MVr strategy remains lacking, especially for intricate valve lesions. Surgeons often perform MVr for acute mitral valve IE primarily based on their prior experience with repair techniques for degenerative mitral valve conditions. It is important to acknowledge that repairs in the context of IE are inherently more intricate and extensive than those for degenerative diseases. In cases of mitral valve endocarditis, the feasibility of repair hinges on the nature of the lesion. The most prevalent lesions, whether in isolation or combination, typically involve leaflet perforation, vegetation, leaflet damage, and chordal rupture. Instances where both the leaflet and the annulus are affected, often accompanied by an annular or paravalvular abscess, are estimated to coexist in approximately 10% to 15% of mitral valve endocarditis cases. This combination has been shown to predict higher mortality rates and increased chances of endocarditis recurrence. When the infection’s extent is more pronounced and the anatomical intricacies are greater, the viability of MVr becomes constrained.

**Figure 1:**
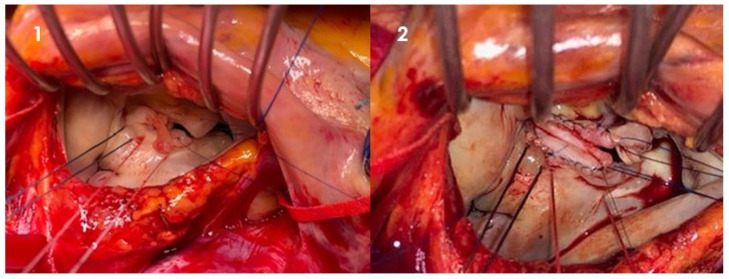
Intraoperative picture of native mitral valve infective endocarditis. Through left atriotomy, exposure of mitral valve. (**1**) Large vegetation involving anterior and posterior leaflet of mitral valve suspended and resected; (**2**) leaflet reconstruction with a pericardial patch (Cardiovascular Department, UO of Cardiac Surgery of IRCCS Humanitas Research Hospital).

In general, techniques may vary between leaflet segmental resection for valve destruction, primary closure, or patch repair (Figure 1) for leaflet perforation and neochordae implantation to replace ruptured chordae and support reconstructed leaflets.

Independently of specific techniques, multiple retrospective trials have proven the effectiveness of MVr in favoring short- and long-term outcomes. Nana Toyoda et al. [13] retrospectively analyzed 1970 patients undergoing isolated MVr (19%) or MVR (81%) for active infective endocarditis between 1998 and 2010. The primary outcome was long-term survival, and the secondary outcomes were recurrent endocarditis and reoperation in the follow-up period. Over the study period, MVr rates increased from 10.7% to 19.4%. Patients undergoing mitral repair tended to be younger, more often in stable hemodynamic conditions, and less likely to have staphylococcal infections. At 12 years, survival was 68.8% after MVr versus 53.5% in the MVR group. MVr was associated with a lower rate of recurrent endocarditis at 12 years (4.7% vs. 9.5%) and a similar rate of reoperation (9.1% vs. 8.6%). Feringa et al. [14] conducted an analysis of 24 studies, revealing that the rates of both early and late mortality after mitral valve repair (MVr) stood at only 2% and 8%, respectively. Following MVr for infective endocarditis (IE), freedom from reoperation exceeded 90% at the 5-year mark and remained above 85% at the 10-year milestone. These findings underscore the sustained durability of MVr over the long term, with consistent results reported across various studies. In a comprehensive assessment by Kang He et al. [15], encompassing a collective of 3759 patients across 17 pertinent publications, 1180 of these patients had undergone MVr, while 2579 had undergone mitral valve replacement (MVR). The patients who opted for MVr experienced a reduced risk of early mortality (RR, 0.51; 95% confidence interval (CI), 0.39–0.66; *p* < 0.00001), a higher rate of long-term survival (HR, 0.69; 95% CI, 0.58–0.81; *p* < 0.001), and a decreased likelihood of recurrence. Additionally, the decisions made prior to surgery, including the timing of the procedure and the rationale for surgical intervention, exhibited a correlation with the percentage of repair procedures performed and their favorable long-term effects. Essentially, the opportunity to undertake a repair technique for native valve endocarditis seems to be linked to the timing of the surgical intervention. Notably, data from North America indicated that the rate of repair was approximately 40–50% for resolved cases of IE but notably lower at 10–20% for acute cases [16]. In a parallel study, Kang et al. [17] conducted a randomized investigation involving IE patients to compare outcomes between an early surgery group and a conventional treatment group. In the early surgery group, where surgery was performed within 2 days of randomization, the rate of mitral valve repair was only 36%, while it reached 68% in the conventional treatment group.

The aortic valve is affected in 35–39% of cases [9]. According to data from the EUROENDO Registry [18], surgical procedures were performed during hospitalization for 1596 patients, constituting 51.2% of the entire cohort. Among these cases, aortic valve procedures were carried out in 54.5% of instances, with a mere 2.3% undergoing aortic valve repair. In recent years, aortic valve repair has progressively emerged as a novel alternative to aortic valve replacement for addressing stable aortic regurgitation (AR). Aortic valve reconstruction has demonstrated not only the ability to circumvent the drawbacks of anticoagulation in younger populations but has also shown a reduced incidence of valve-related complications when compared to previously reported rates associated with aortic valve replacement. Unlike in mitral valve diseases, the potential for aortic valve repair is limited and technically demanding. Additionally, within the context of endocarditis, aortic valve repair is even less established. One of the main reports on this topic is from Mayer et al. [19]. Their study retrospectively analyzed a cohort of 100 patients diagnosed with active native aortic valve infective endocarditis (IE). Among them, 33 patients underwent aortic valve repair (AVr), while 67 patients opted for aortic valve replacement (AVR). In terms of the absence of Grade II or higher recurrent aortic regurgitation, freedom from thromboembolic events at 5 years, and recurrence of endocarditis, no significant disparities were observed between the two groups. However, the survival rate at 4 years was notably superior following repair. The actuarial freedom from reoperation after 5 years stood at 65% in the AVr group, as opposed to 90% in the AVR group. The researchers also established a correlation between the size of patches used and the risk of late failure. AVr has demonstrated its worth as a viable substitute for aortic valve replacement (AVR) in case of aortic insufficiency, even with bicuspid aortic valves [20,21]. Research conducted by Mayer et al. further pointed out that AVr can be considered a feasible option in active infective endocarditis, albeit in exceedingly carefully chosen patients and in very expert skilled hands. It should be mentioned that a possible alternative to valve replacement has been described by Ozaki et al. They created a technique for aortic valve reconstruction at Toho University Ohashi Medical Center, where they established a standardized approach employing glutaraldehyde-treated autologous pericardium for trileaflet aortic valve neocuspidization (AVneo). In 2018, the Ozaki team documented their comprehensive group of 850 patients. The study revealed that the observed survival rate, freedom from reoperation, and the occurrence of moderate or greater aortic regurgitation were 85.9%, 95.8%, and 7.3%, respectively, over an average follow-up period of 4.4 years. Within this patient cohort, 19 cases reported an infective endocarditis diagnosis; however, the outcomes specific to this subgroup were not detailed [22]. Aortic valve infective endocarditis (IE) has the potential to extend its infection beyond the valve leaflets, presenting a complex surgical scenario. When a straightforward valve replacement is necessary, the selection of prosthesis should adhere to standard criteria. Conversely, the determination of which valve substitute to employ for the reconstruction of the left ventricle outflow tract (LVOT), and which option offers superior survival, function, and reduced reinfection rates, remains a topic of ongoing discussion. Among the surgical options available to these patients are aortic valve replacement (AVR) using bioprosthetic or mechanical valves, either with or without patch support; stentless valves; aortic allografts; or composite valve grafts. In cases involving paravalvular abscess, the complete removal of all infected tissues is a crucial step before deciding on a suitable valve substitute. If aorto—ventricular dehiscence occurs after thorough debridement, aortic root replacement becomes a necessary intervention. More intricate settings influence surgical scenarios and subsequent survival outcomes, which can introduce complexity when attempting a statistical comparison. From a durability standpoint, the rates of survival and freedom from reoperation for homografts are akin to those observed with bioprosthetic valves, although they are inferior to the durability exhibited by mechanical valves [23]. In a study by El Hamamsy et al. [24], a randomized trial involving aortic root replacement utilizing either a homograft or the Freestyle stentless valve (Medtronic Inc, Minneapolis, MN, USA) demonstrated a minor, yet significant, decrease in freedom from reoperation at the 8-year mark for the homograft group (90% versus 100%) while exhibiting a comparable 8-year survival rate.

Klieverik et al. [25] found no discernible difference in survival rates at the 8-year or in instances of late recurrent endocarditis, but they observed a significantly elevated rate of freedom from reoperation at the 15-year mark for mechanical prostheses compared to homografts (93% versus 76%; *p* = 0.002) among younger patients. This result implies that younger patients experience a greater advantage in terms of freedom from reoperation when opting for mechanical prostheses.

**Figure 2:**
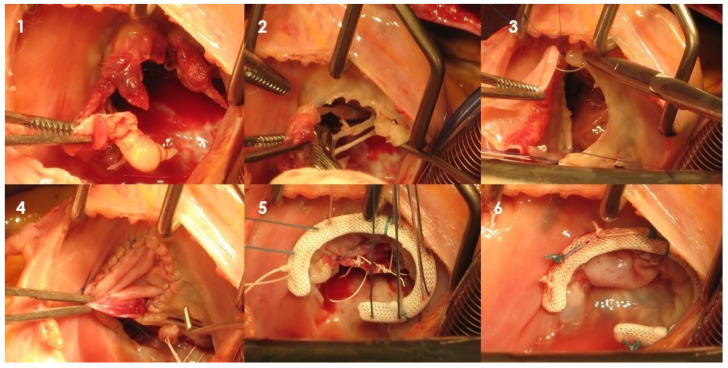
Intraoperative picture of native tricuspid valve infective endocarditis. Through right atriotomy, exposure of tricuspid valve. (**1**,**2**) Large vegetation is detected and resected on the anterior leaflet; (**3**,**4**) reconstruction of the leaflet is obtained through a pericardial patch; (**5**) neochordal apparatus is sutured on the free margin of the new anterior leaflet, and in order to optimize coaptation and competence, an incomplete tricuspid annular ring is positioned; (**6**) final result (Cardiovascular Department, UO of Cardiac Surgery of IRCCS Humanitas Research Hospital).

Compared to left-sided infective endocarditis (IE), the incidence of right-sided IE tends to be lower, possibly due to the relatively infrequent prevalence of typical pathological conditions affecting the right-sided valves. This variation may stem from differences in the properties of the right-sided endothelium and disparities in right-side hemodynamic patterns, characterized by lower pressure gradients and jet velocities across the right-sided valves, as well as reduced right-sided wall stress and lower oxygen content in venous blood [26]. Over the past two decades, there has been a steady increase in the occurrence of tricuspid valve (TV) IE. This rise is attributable to the growing prevalence of factors such as intravenous drug use, the implantation of right-sided cardiac devices, central venous catheterization, and repaired congenital defects [27]. Similar to what has been emphasized regarding mitral valve IE, the guiding principles for surgical intervention in TV IE continue to favor repair over replacement. This approach helps mitigate the risks of prosthetic valve deterioration and lowers the likelihood of recurrence. A variety of techniques have already been described in the case of functional tricuspid regurgitation. Tricuspid valve repair (TVr) can be achieved using several methods (Figure 2), including vegectomy, bicuspidization, leaflet patch application, chordal replacement, and prosthetic ring annuloplasty. The advantage of TVr lies in its limited use of foreign materials, which, in turn, reduces the risk of reinfection. Additionally, it may obviate the need for anticoagulation therapy and carries a lower risk of heart block, thereby decreasing the potential for requiring a permanent pacemaker. The population affected by tricuspid valve IE frequently includes individuals with a history of intravenous drug use. In these patients, it is particularly crucial to mitigate the risk of recurrent infection due to the heightened vulnerability to relapse and subsequent IE, compounded by a shorter overall life expectancy.

The outcomes of tricuspid valve repair (TVr) and tricuspid valve replacement (TVR) are indeed comparable in terms of long-term survival. Yanagawa B. et al. [28] conducted an analysis of 12 unmatched retrospective observational studies encompassing 1165 patients. Their goal was to compare the early and late outcomes of tricuspid valve repair versus replacement. The primary indications for surgery included septic pulmonary embolism, left-sided infective endocarditis (IE), right-sided heart failure, and persistent bacteremia. Across the studies, the median proportion of repairs was 59%, while replacements constituted 41%. Common repair strategies included vegetectomy, the De Vega procedure, annuloplasty ring insertion, bicuspidization, and leaflet patch augmentation. Among valve replacements, bioprosthetic valves accounted for 83%, while mechanical prostheses comprised 17%. The analysis revealed no significant differences between TVr and TVR in terms of perioperative mortality and long-term all-cause mortality. However, TVr was linked to lower rates of recurrent IE and a reduced need for reoperation, although a trend toward a greater risk of moderate to severe tricuspid regurgitation was noted. Additionally, TVr was associated with a decreased requirement for a permanent pacemaker. Lee et al. [29] conducted a comprehensive nationwide population-based cohort study involving 704 patients from the Taiwan National Health Insurance Research Database. These patients had undergone tricuspid valve surgery due to infective endocarditis between 2000 and 2013. The study indicated that the in-hospital mortality rate between the TVr and TVR groups did not exhibit a significant difference. However, TVr demonstrated lower rates of perioperative complications, including factors such as massive blood transfusion, initiation of dialysis, and deep wound infections. Patients who underwent TVr experienced longer stays in the intensive care unit and the hospital, along with higher hospital costs. Furthermore, TVr was linked to a decreased risk of re-admission, new permanent pacemaker implantation, and all-cause mortality in comparison to TVR. A recent series of cases focused on patients who were intravenous drug abusers and diagnosed with tricuspid valve infective endocarditis. Slaughter et al. [30] conducted an investigation involving 1613 patients in this category who underwent isolated tricuspid valve procedures due to endocarditis. The patients were categorized based on the type of operation performed: valvectomy (7%), tricuspid valve repair (TVr, 33%), and tricuspid valve replacement (TVR, 60%). The purpose of this categorization was to assess risk factors and 30-day outcomes. The valvectomy group exhibited a higher frequency of acute infection (90% compared to 79% and 84%), a higher Model for End-Stage Liver Disease (MELD) score (10.17% compared to 8.44% and 9.74%), and a higher incidence of urgent/emergent surgeries (91% compared to 75% and 83%). Notably, the operative mortality rate was notably higher in the valvectomy group (16%) as opposed to the TVr (2%) or TVR (3%) groups. Based on their study, valvectomy emerged as an independent predictor of operative mortality. The authors recommend, when feasible from an anatomical perspective, prioritizing TVr to minimize the risk of recurrent valve infection and prosthetic valve degeneration. It is worth mentioning that pulmonic valve infective endocarditis (IE) can coexist with tricuspid valve IE [31]. Isolated pulmonic valve IE is rare, accounting for less than 2% of IE cases [32]. Chowdhury et al. reviewed all cases of isolated pulmonary valve endocarditis published between 1979 and 2013, identifying only 70 reported cases [33]. Hussain et al. studied patients who underwent surgery for active IE (138 right-sided and 1224 left-sided cases), and among them, only 7 exhibited pulmonic valve involvement [34].

In cases of right-sided IE, an alternative to surgery involves the utilization of percutaneous, vacuum-assisted devices designed for removing intracardiac masses. Such devices serve as a means to remove large tricuspid valve vegetations when the risks associated with surgery are prohibitive. In 2014, the US Food and Drug Administration approved the AngioVac system (AngioDynamics, Latham, NY, USA), a device designed for removing undesired intravascular materials, such as thrombi and emboli [35]. George et al. [36] retrospectively analyzed 33 patients with tricuspid valve IE in which a percutaneous aspiration device was employed to manage large vegetations. Reductions in vegetation size were observed in 61% of cases, and 91% of patients were discharged home. Other reports, such as those by Patel et al. [37], suggest that the use of percutaneous aspiration devices prior to percutaneous-lead extraction may lower the incidence of septic pulmonary embolism. However, it is important to acknowledge that percutaneous vacuum-assisted devices come with their own set of potential adverse risks, including the embolization of vegetations into pulmonary circulation and complications related to vascular access, such as bleeding and the potential for infection. Abubakar et al. [38] reported that the risk of septic pulmonary embolism remains significant, ranging from 34% to 55%, particularly in patients with vegetations larger than 1 cm. As of now, there has been no official prospective comparison of an AngioVac device with either medical therapy or surgical intervention, and no guidelines have yet been established in this regard.

## 3. Prosthetic Valve and Implantable Device Endocarditis

The epidemiology of infective endocarditis is changing and the incidence of prosthetic valve (PV) IE is increasing, as proven by the comparison of the different registries provided through the years. According to the latest, PV IE accounts for 30% of cases in the EURO-ENDO Registry, 25% in the 2008 French registry, and 21% in the International Collaboration on Endocarditis Prospective Cohort Study reported in 2009 [8,18,39,40]. Prosthetic valve endocarditis represents a significant complication of valve replacement, occurring at a rate of 0.3% to 1.2% per patient-year. This corresponds to approximately 3% to 6% of individuals who receive a prosthetic valve experiencing this complication within the first 5 years following implantation [41]. Patients undergoing surgery for prosthetic valve infective endocarditis (PV IE) present a diverse range of clinical scenarios, often being older and more prone to nosocomial infections. In comparison to cases of native valve endocarditis, PV IE continues to be a severe condition associated with notably higher in-hospital mortality rates, ranging from 19% to 50%, as opposed to the 7% to 13% rates observed in patients with native valve IE. If not addressed promptly, PV IE can lead to severe anatomical consequences such as valvular apparatus destruction, abscess formation, pseudoaneurysms, fistulas, perforations, heart block, and strokes. Della Corte et al. [41] documented 30-day mortality rates of around 20% for patients undergoing surgery for PV IE across three distinct eras. Similarly, Lalani et al. [42] reported comparable rates, attributing these findings to various factors, including advanced patient age, the presence of Staphylococcus aureus infections, and infections acquired within healthcare settings. The observed higher early mortality rate might also stem from the technically more challenging nature of interventions in cases of PV IE. In support of this, Alonso-Valle et al. [43] noted the highest mortality rate during the initial 3 months following hospital discharge, with patient survival stabilizing thereafter. Weber et al. [44] retrospectively analyzed 4300 patients undergoing valve surgery for NV IE or PV IE taking data from 5 German Cardiac Surgery Centers. Patients with prosthetic valve endocarditis were older and had more comorbidities, so the Kaplan–Meier analysis revealed significantly decreased long-term survival of patients with PV IE compared with those operated for NV IE. It should be noted that after multivariable adjustment, there was no significant difference in long-term survival.

Both mechanical and bioprosthetic valves are susceptible to infection, exhibiting no significant difference in prevalence within the first 5 years (5.7%). Interestingly, mechanical valves present a heightened risk of infection during the initial 3 months following operation [45], whereas bioprosthetic valves demonstrate an elevated risk at the 18-month mark post valve replacement [46]. The population of pathogens that contribute to infective endocarditis (IE) in prosthetic valves varies depending on whether the period being considered is within 1 year from the operation or beyond. This one-year timeframe is conventionally used as a cutoff to differentiate early and late prosthetic valve IE [47]. Instances of early prosthetic valve IE (occurring within the first year) are most prevalent in the initial two months following valve replacement. This is attributed to microorganisms that either invade the prosthesis during the surgical intervention or disseminate hematogenously in the early days or months post-surgery. Notably, a similar microbiologic profile has been observed between cases of prosthetic valve IE within 2 months of valve replacement and those occurring between 2 and 12 months after the surgery [48]. In the initial year following valve replacement, both aortic and mitral valve prostheses are affected equally, and there is no difference in the involvement of mechanical and biological prostheses [49].

Additionally, a noteworthy and emergent concern in the context of prosthetic valve IE involves infections associated with transcatheter-implanted valves and percutaneous edge-to-edge mitral valve repair. Incidences of IE following transcatheter aortic valve implantation (TAVI) appear to be relatively low, with certain studies reporting around 1% incidence [50], albeit being slightly higher among male patients. However, in studies with longer follow-up periods and larger patient cohorts, the occurrence of infective endocarditis is more notable, with a cumulative incidence of TAVI prosthesis infection reaching up to 5% during the initial 5 years [51]. The majority of TAVI endocarditis cases (90%) are managed conservatively, resulting in high in-hospital mortality and suboptimal short-term survival [52]. Surgical approaches involving TAVI prosthesis explantation have not demonstrated superior survival rates, although such reports are scarce. This specific context still presents limited experience with treatment strategies, and a significant number of patients are often considered inoperable or at high surgical risk in elective settings. As indications for TAVI expand to encompass intermediate- to low-risk patients, a comprehensive understanding of TAVI endocarditis treatment, comparative evaluations of medical versus surgical options, and well-defined surgical treatment pathways are essential. Conversely, the risk of IE in patients undergoing percutaneous edge-to-edge mitral valve repair using the MitraClip (Abbott, Abbott Park, IL, USA) appears to be quite low. A comprehensive registry of MitraClip patients did not document a single instance of IE throughout a 5-year follow-up period [53].

## 4. Surgery in Complex Infective Endocarditis: Current Trends

**Figure 3:**
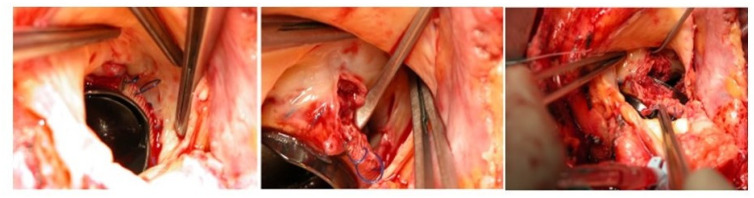
Intraoperative picture of mechanical prosthetic aortic valve endocarditis and aortic annular erosion and abscess, detected during the prosthesis removal (Cardiovascular Department, UO of Cardiac Surgery of IRCCS Humanitas Research Hospital).

Endocarditis is a potentially destructive disease and the treatment can widely range in complexity from medical antimicrobial treatment to complex cardiac reconstruction. Endocarditic lesions may manifest as small vegetations originating from the cusps or leaflets, or fenestrations, which can often be easily treated with excision and valve replacement or valve repair, as already mentioned. If the disease progresses locally, it may lead to valvular annulus involvement and the development of an annular abscess. In turn, a deeper myocardial involvement may lead to damage to the fibrous skeleton of the heart and to the conduction system, as well as perforation into other cardiac chambers. The scope and nature of surgical intervention are directly contingent upon the extent of tissue damage, consistently adhering to the principle of thorough debridement, and may necessitate annular reconstruction. The choice of material for the new annulus is generally a matter of surgeon preference, with options including bovine pericardium, both fixed and fresh autologous pericardium, as well as Dacron, all being documented choices. Pericardial tissue holds the advantage of increased pliability and the capacity to conform to the underlying myocardium, thereby reducing the likelihood of flow beneath the patch that could potentially lead to the development of a pseudoaneurysm or shunt. Additionally, the utilization of autologous or heterologous pericardium has not demonstrated an elevated risk of reinfection. Following the placement of the patch, the decision between valve repair and replacement is determined by the extent of valve leaflet or cusp destruction. Approximately 15% of cases of mitral valve infective endocarditis (MVE) may involve a mitral annular abscess [54]. These abscesses are typically located in the posterior mitral annulus, encompassing the posterior leaflet, the annulus itself, and the underlying myocardium. When an annular abscess is present or substantial destruction of the posterior annulus is evident, this implies the necessity of debridement and subsequent reconstruction of the atrioventricular groove using a patch, prior to proceeding with mitral valve surgery. The patch aims to reconstruct atrioventricular continuity and should always be oversized in order to comfortably cover the defect circumferentially. Attention must be paid to the underlying left circumflex coronary artery. Oversizing the patch guarantees good sealing, thanks to the intracavitary ventricular pressure against the myocardium, and contributes to hemostasis. Aortic annular erosion and abscess can be detected in both native and prosthetic aortic valve endocarditis (Figure 3).

**Figure 4:**
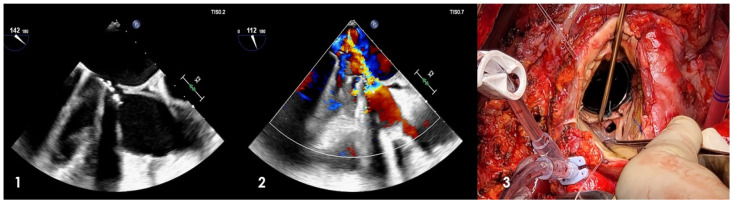
Aortomitral curtain disjunction in double mechanical prosthesis infective endocarditis. (**1**,**2**) Transesophageal echocardiogram imaging showing communication and jet from the ascending aorta to the left atrium; (**3**) intraoperative founding of aortomitral curtain disjunction detected through the aortotomy (*black hole*) (Cardiovascular Department, UO of Cardiac Surgery of IRCCS Humanitas Research Hospital).

Managing such lesions can be intricate due to extensive tissue damage, limited available area for prosthesis implantation, and, in more severe cases, potential left ventricular aortic discontinuity. Surgical intervention involves the thorough removal of the infected region and rectification of the annular defect. Multiple techniques have been outlined to address this condition. Typically, closure of the abscess cavity involves the implementation of a patch to rebuild the left ventricle outflow tract, followed by valve or root replacement. In instances where profound circumferential destruction of the aortic annulus is observed, some experts propose an alternative approach involving the reconstruction of the left ventricle outflow tract and the translocation of the aortic valve into the ascending aorta. This technique aims to position the new valve prosthesis away from the infected area [55]. According to the EuroEndo analysis, the prevalence of multiple-valve endocarditis stands at approximately 18.2% among all patients. Invasive double-valve endocarditis, affecting both the aortic and mitral valves, entails the compromise of the fibrous heart skeleton and necessitates intricate surgical management involving higher risk. As infective endocarditis advances through the aortic and mitral valves, it erodes the aortic mitral curtain, leading to its destruction and the subsequent development of abscesses, pseudoaneurysms, or fistulas (Figure 4).

The aortic mitral curtain (AMC) is a complex component of the heart’s fibrous skeleton, situated between the lateral and medial trigones of the mitral valve. It forms connections between the left and non-coronary cusps of the aortic valve and links the anterior leaflet of the mitral valve to the aortic valve cusps and the roof of the left atrium, effectively separating the mitral valve from the aortic root. Given its invasive nature, managing this structure typically necessitates a complex surgical procedure. Initially proposed by Tirone David, the surgical approach involves double valve replacement using pericardial or Dacron patches to reestablish “neoaortomitral continuity” [56]. This procedure has been termed the “Commando procedure” by the Cleveland Clinic group and referred to as the “UFO procedure” by the Leipzig group [57]. For cases in which the infection has not affected the posterior mitral leaflet and at least the free edge of the anterior mitral leaflet remains viable, a modified version of the Commando procedure, known as the “Hemi-Commando procedure”, has been suggested [58]. The Commando procedure, being an invasive surgical option, offers several advantages in cases of infective endocarditis (IE). It ensures the complete removal of infected material and reduces the risk of prosthesis dehiscence, paravalvular leaks, and patient–prosthesis mismatch, all of which could lead to the need for re-intervention [59]. However, comparing the outcomes of the Commando procedure to less aggressive strategies, both in terms of mortality rates and the risk of re-intervention, is challenging due to the rarity of the disease, the generally poor health of patients requiring this procedure, and the extensive involvement and damage to cardiac structures. It is widely recognized that this aggressive approach should be considered whenever feasible, particularly under the care of experienced surgeons. A study by Darvierwala et al. [60] demonstrated that with increasing surgical experience, the highest mortality occurs within the first 90 days post-surgery, after which survival rates improve to around 70%. Long-term mortality rates range from 60% to 70% at the 5-year mark, and this is not significantly different when compared to IE patients treated using standard approaches. [61]. Croon SI et al. [62] showed a survival rate of 55% at 10 years. Irrespective of the surgical technique, which directly depends on the anatomical scenario, another important key step is the choice of the prosthesis or conduit. Currently, existing guidelines do not advocate for the prioritization of one substitute over another, and the optimal choice of valve type remains more influenced by individual surgical expertise than standardized guidelines. The available substitutes encompass homografts, autografts, stented or non-stented xenograft prostheses, and mechanical prostheses. Within the context of infective endocarditis, the advantages of utilizing biological prostheses are well established. Although evidence suggests that opting for a biological valve instead of a mechanical prosthesis offers additional benefits in terms of long-term outcomes, the efficacy and safety of each prosthesis type are intertwined with the patient’s age and the implant location [63,64]. In the mitral position, mechanical prostheses are a viable choice for patients up to the age of 70 [65], while in the aortic position, the advantages of mechanical prostheses diminish after the age of 55 [65]. The benefits of lower reoperation risk associated with mechanical valves need to be weighed against the higher risk of bleeding and stroke in specific age groups [66]. Evidence indicates that selecting a homograft or even an autograft proves advantageous for younger high-risk patients, particularly in cases of complex valve endocarditis or recurrent prosthetic valve infective endocarditis (PV IE). The use of homografts is particularly advantageous within the first ten years post-surgery due to the reduced risk of recurrent infection [67,68]. However, over time, the risk of structural valve deterioration increases [69]. The choice of graft type should be informed by various factors including the patient’s age, extent of infection (especially involving the mitral valve), engagement of other cardiac structures, and susceptibility to infections.

## 5. Conclusions

The literature and guidelines on the surgical management of infective endocarditis are based mainly on observational studies, given the difficulty in designing randomized trials in such a complex setting often present in urgent contexts. Due to the varying anatomical and clinical scenarios, heart teams are often brought to difficult decisions on the most appropriate strategy to adopt; moreover, the best choices are made through a shared decision-making process that should include the patient too. Decision making should take into account the localization and extent of the infection, patient preoperative status and comorbidities, and the choice of the best timing for surgery. Special relevance should be given to timing, which influences the risk of neurological complications and the extent of the infection, which, in turn, have a strong impact on post-operative outcomes and mortality rates. Whenever possible, surgeons should prefer a repair strategy over replacement in order to avoid the insertion of prosthetic material into infected tissue, limit the extent of the infection process, and better preserve ventricular function. Another important key step is the choice of the prosthesis or conduit. To date, guidelines do not support the selection of one substitute over another, and the selection of the most appropriate valve replacement strategy or conduit should consider the longevity of the substitutes, especially when biological, the potential recurrence of infection, and the risk of redo surgery. Surgeons should be able to maneuver and master all the reconstructive techniques described in the literature in order to manage all possible anatomical and pathological settings, particularly in consideration of the recent and future rise in cardiovascular device implantation and the consequent need for removal in case of endocarditis.

## Figures and Tables

**Table 1:** Comparison based on the main surgical indications according to European and American guidelines in treatment of infective endocarditis [4,5].

European Society of Cardiology (ESC) Guidelines 2015	American Association for Thoracic Surgery (AATS) Guidelines 2016
**Heart Failure**	
Aortic or mitral native valve endocarditis (NVE) or prosthetic valve endocarditis (PVE) with significant acute regurgitation, obstruction, or fistula leading to cardiogenic shock (EMERGENCY, I, B) or exhibiting inadequate hemodynamic tolerance (URGENT, I, B).	Surgery when patients with IE present with valve dysfunction, resulting in symptoms of heart failure (I, B).
**Uncontrolled Infection**	
Locally uncontrollable infection requiring immediate attention (URGENT, I, B); infection stemming from fungi or highly resistant organisms (URGENT/ELECTIVE, I, C); continued presence of positive blood cultures, despite suitable antibiotic treatment and effective management of septic secondary sites (URGENT, IIa, B); prosthetic valve endocarditis (PVE) resulting from staphylococci or non-HACEK Gram-negative bacteria (URGENT/ELECTIVE, IIa, C).	Surgical intervention is recommended for individuals with left-sided infective endocarditis (IE) triggered by S. aureus, fungal agents, or other extensively resistant microorganisms (I, B). Surgery is also advisable when there are destructive penetrating lesions present (I, B) or in cases of persistent bacteremia or fever that extend beyond 5 to 7 days, despite the initiation of suitable antimicrobial treatment (I, B). Cases of prosthetic valve endocarditis (PVE) and recurring infections (IIa, C).
**Prevention of Embolism**	
Aortic or mitral native valve endocarditis (NVE) or prosthetic valve endocarditis (PVE) characterized by persistent vegetations exceeding 10 mm size after one or more embolic episodes, despite the administration of suitable antibiotic therapy (URGENT, I, B). In cases of severe valve stenosis or regurgitation with low surgical risk (URGENT, I, B). For aortic or mitral NVE or PVE featuring isolated very large vegetations (greater than 30 mm) (URGENT; IIa, B). In situations where aortic or mitral NVE or PVE presents with isolated large vegetations (over 15 mm) and no other evident indications for surgery (URGENT, IIb, C).	Existence of repeated embolic events and persistent vegetations, despite suitable antibiotic treatment (IIa, B); cases of native valve endocarditis (NVE) or prosthetic valve endocarditis (PVE) characterized by mobile vegetations exceeding 10 mm length, accompanied by clinical signs of embolic occurrences even after appropriate antimicrobial therapy, warrant immediate or urgent attention (IIb, B).
**Right-sided Infective Endocarditis**	
Microorganisms that are hard to eliminate or cases where bacteremia persists > 7 days, despite appropriate antimicrobial treatment; tricuspid valve vegetations that persist beyond 20 mm following recurrent pulmonary emboli, regardless of the presence or absence of concurrent right heart failure or right HF triggered by severe TR (IIa, C).	Native valve endocarditis (NVE) or prosthetic valve endocarditis (PVE), surgery if there is evident symptomatic and severe valve malfunction, sizable vegetations, or persistent infection indicated by continuous bacteremia or prolonged fever lasting beyond 5 to 7 days, despite appropriate antimicrobial treatment, or if septic pulmonary embolism (IIb, B).

## Data Availability

No new data were created or analyzed in this study. Data sharing is not applicable to this article.
